# A plasmid with the blaCTX-M gene enhances the fitness of Escherichia coli strains under laboratory conditions

**DOI:** 10.1099/mic.0.001525

**Published:** 2025-01-30

**Authors:** Lázaro López, Diana Calderón, Liseth Salinas, Jay P. Graham, Zachary D. Blount, Gabriel Trueba

**Affiliations:** 1 Instituto de Microbiología, Colegio de Ciencias Biológicas y Ambientales, Universidad San Francisco de Quito, Quito, Ecuador; 2 Laboratorio de Biotecnología de Plantas, Universidad San Francisco de Quito, Quito, Ecuador; 3 Environmental Health Sciences Division, University of California, Berkeley, California, USA; 4 Department of Microbiology, Genetics, and Immunology, Michigan State University, East Lansing, Michigan, USA

**Keywords:** antibiotic-resistant burden, *bla*_CTX-M_, ESBL fitness cost, *Escherichia coli*, plasmid fitness cost, plasmid maintenance

## Abstract

Antimicrobial resistance (AMR) is a major threat to global public health that continues to grow owing to selective pressure caused by the use and overuse of antimicrobial drugs. Resistance spread by plasmids is of special concern, as they can mediate a wide distribution of AMR genes, including those encoding extended-spectrum *β*-lactamases (ESBLs). The CTX-M family of ESBLs has rapidly spread worldwide, playing a large role in the declining effectiveness of third-generation cephalosporins. This rapid spread across the planet is puzzling given that plasmids carrying AMR genes have been hypothesized to incur a fitness cost to their hosts in the absence of antibiotics. Here, we focus on a WT plasmid that carries the *bla*_CTX-M 55_ ESBL gene. We examine its conjugation rates and use head-to-head competitions to assay its associated fitness costs in both laboratory and wild *Escherichia coli* strains. We found that the wild strains exhibit intermediate conjugation levels, falling between two high-conjugation and two low-conjugation laboratory strains, the latter being older and more ancestral. We also show that the plasmid increases the fitness of both WT and lab strains when grown in lysogeny broth and Davis–Mingioli media without antibiotics, which might stem from metabolic benefits conferred on the host, or from interactions between the host and the rifampicin-resistant mutation we used as a selective marker. Laboratory strains displayed higher conjugation frequencies compared to WT strains. The exception was a low-passage K-12 strain, suggesting that prolonged laboratory cultivation may have compromised bacterial defences against plasmids. Despite low transfer rates among WT *E. coli*, the plasmid carried low fitness cost in minimal medium but conferred improved fitness in enriched medium, indicating a complex interplay between plasmids, host genetics and environmental conditions. Our findings reveal an intricate relationship between plasmid carriage and bacterial fitness. Moreover, they show that resistance plasmids can confer adaptive advantages to their hosts beyond AMR. Altogether, these results highlight that a closer study of plasmid dynamics is critical for developing a secure understanding of how they evolve and affect bacterial adaptability that is necessary for combating resistance spread.

**Impact statement**

This study investigates the impact of a plasmid carrying the *bla*_CTX-M-55_ gene on its *Escherichia coli* host, with the goal of elucidating its role in the spread and persistence of third-generation cephalosporin resistance. By analysing the fitness effects of antimicrobial resistance plasmids, this research offers critical insights into the evolutionary dynamics of antibiotic resistance and its interactions with bacterial hosts. Improving our understanding of how plasmids affect host fitness is vital for developing effective strategies to combat the spread of antimicrobial resistance within microbial populations. Given the increasing prevalence of *bla*_CTX-M_ through plasmids, a deeper comprehension is essential for addressing the growing threat of antimicrobial resistance [82].

## Introduction

The spread of antimicrobial resistance (AMR) has increased rates of illness and death worldwide and placed growing economic strain on healthcare systems, making it the foremost threat to global public health [1,2]. Global deaths attributable to AMR grew from 700 000 in 2016 to 1.2 million in 2019 and are projected to grow to 10 million per year in 2050 [3,4]. Indeed, AMR is now responsible for more deaths than most major infectious diseases, including malaria and Human Immunodeficiency Virus (HIV)/AIDS [3]. The incidence of AMR infections that do not lead to death is orders of magnitude higher still, exacerbating the associated economic impact. Indeed, a single pathogenic AMR *Escherichia coli* lineage, designated as sequence type 131, is alone responsible for millions of infections globally each year [2].

The spread of AMR has been driven by the use and overuse of antimicrobial drugs and the pervasive and strong selection of resistant microbes that it has created. Consequently, these drugs have lost much of their effectiveness over time, which has made once-treatable infections more difficult to manage. Expanded-spectrum cephalosporins (ESCs), a class of broad-spectrum *β*-lactam antibiotics, are a typical example of this dynamic. When ESCs were introduced in the early 1980s, they marked a major advance in the treatment of infections caused by *Enterobacterales* and other Gram-negative pathogens [5,6]. However, the widespread use of *β*-lactam antibiotics led to the rapid spread of genes for extended-spectrum *β*-lactamases (ESBLs), which confer resistance by hydrolysing the *β*-lactam ring [7,8].

Over 200 ESBLs have been identified, most of which derive from 2b *β*-lactamases that have accrued mutations that have broadened their substrate specificity to include ESCs [5]. Major groups include *bla*_TEM_, *bla*_SHV_, *bla*_OXA_ and *bla*_CTX-M_; the latter is the predominant gene responsible for ESC resistance [9,10]. The *bla*_CTX-M-55_ gene is of particular interest because it has rapidly spread worldwide since it was first discovered in 2004 [11] and it is a major gene found in Ecuadorian isolates [12]. This gene belongs to the CTX-M-1 group and differs in only a single aa from *bla*_CTX-M-15_, which is also being closely studied owing to its role in ESC resistance [11]. As with other genes for AMR, those for ESBLs are mobilized by plasmids or other mobile genetic elements. These plasmids often carry other genes that confer resistance to diverse drug classes, including sulphonamides, aminoglycosides, fluoroquinolones and the novel phosphonic antibiotic fosfomycin [13,16].

Plasmids, including those involved in AMR, have long been hypothesized to impose fitness burdens on their host bacteria [17]. This hypothesized cost has led theorists to predict that plasmids must engage in rapid horizontal spread to avoid elimination by host populations [18]. AMR plasmids should therefore be rapidly lost in the absence of the antibiotics to which they confer resistance [19].

Despite these predictions, AMR plasmids can persist in bacterial populations for extended periods of relaxed antibiotic selection [20,22]. This widespread occurrence and persistence of AMR plasmids has been referred to as the ‘plasmid paradox’. A number of hypotheses have been proposed to explain the paradox by invoking various phenomena and mechanisms, including plasmid-conferred traits that are more generally adaptive to the host (e.g. metabolic improvements), and compensatory mutations that alleviate fitness costs and drive host–plasmid co-adaptation [23,26].

Studies have shown that plasmids can have surprisingly variable fitness effects on their bacterial hosts [20]. However, it is currently unclear how common this variation might be among and across different plasmids and hosts. Here, we examine plasmid p201809181.3. Originally isolated from an Ecuadorian child, p201809181.3 is a clinically relevant, multireplicon plasmid that carries *bla*_CTX-M-55_ and is related to pHN7A8, a chimeric, multidrug resistance plasmid first identified in China [12,27, 28]. The *bla*_CTX-M-55_ gene is accompanied by the *bla*_TEM_ and *fosA3* genes, which together form a resistance cassette that is flanked by IS26 insertion sequences [27]. This combination of genes grants host bacteria multidrug resistance to penicillins, cephalosporins and fosfomycin. To better understand the dynamics and persistence of p201809.3, we examined its conjugation rate and fitness effects across a collection of *E. coli* strains, which includes isolates from natural communities in Ecuador as well as standard laboratory strains.

## Methods

### Bacterial strains

Plasmid p201809181.3 was originally discovered in *E. coli* 201809181.3, a commensal strain that was isolated from a human stool sample [12]. We conjugated p201809181.3 into a collection of recipient strains, which includes both standard laboratory strains and natural isolates of *E. coli*. The laboratory strains were Crooks (ATCC 8739), K-12 (NCTC 10538), the high-efficiency cloning strain, TOP10, and J53, a sodium azide-resistant derivative of K-12 commonly used in conjugation experiments [29]. The natural isolates, W1 and W2, are antibiotic-sensitive commensal strains cultured from faecal samples taken from healthy donors [30]. We also used *E. coli* strain 14.SA.05, which is unable to ferment lactose and was isolated from a healthy donor’s faecal sample, as a common competitor to assess the fitness of strain W1, with and without p201809181.3 [30].

### Spontaneous rifampicin mutant selection

We used a protocol previously described by López *et al.* to isolate spontaneous rifampicin-resistant mutants of each recipient strain for use in conjugation experiments [31]. Briefly, we inoculated recipient strains into 10 ml lysogeny broth (LB) and grew them overnight at 37 °C with 200 r.p.m. orbital shaking speed. We then spread 100 µl of the cultures on MacConkey lactose (MKL) agar supplemented with 100 µg ml^−1^ rifampicin and incubated overnight at 37 °C. We randomly selected one Rif^r^ colony for each recipient and again streaked it to MKL rifampicin agar and incubated overnight at 37 °C. We then inoculated a purified rifampicin-resistant colony into brain heart infusion broth, grew it overnight and froze aliquots with 20% glycerol at −80 °C. These rifampicin-resistant strains were designated as K-12^rr^, Crooks^rr^, W1^rr^ and W2^rr^.

### Antibiotic susceptibility testing

We determined the antibiotic susceptibility profiles for all strains using the disc diffusion method as described by the Clinical and Laboratory Standards Institute (CLSI) guidelines using the following 17 antimicrobial discs: ampicillin (AM; 10 µg; BBL™), fosfomycin (FOS; 200 µg; BD BBL™), streptomycin (S; 10 µg; OXOID), gentamicin (GM; 10 µg; BBL™), kanamycin (K; 30 µg; BIOANALYSE^®^), ciprofloxacin (CIP; 5 µg; BBL™), chloramphenicol (C; 30 µg; BD BBL™), trimethropim-sulphamethoxazole (SXT; 1.25–23.75 µg; BD BBL™), tetracycline (TE; 30 µg; BBL™), azithromycin (AZM; 15 µg; BD BBL™), cefazolin (CZ; 30 µg; BD BBL™), cefuroxime (CXM; 30 µg; BBL™), ceftazidime (CAZ; 30 µg; BBL™), ceftriaxone (CRO; 30 µg; BBL™), cefepime (FEP; 30 µg; BD BBL™), imipenem (IPM; 10 µg; BBL™) and nitrofurantoin (F/M; 300 µg; BBL™) [32].

### Conjugation assays

**Fig. 1.**
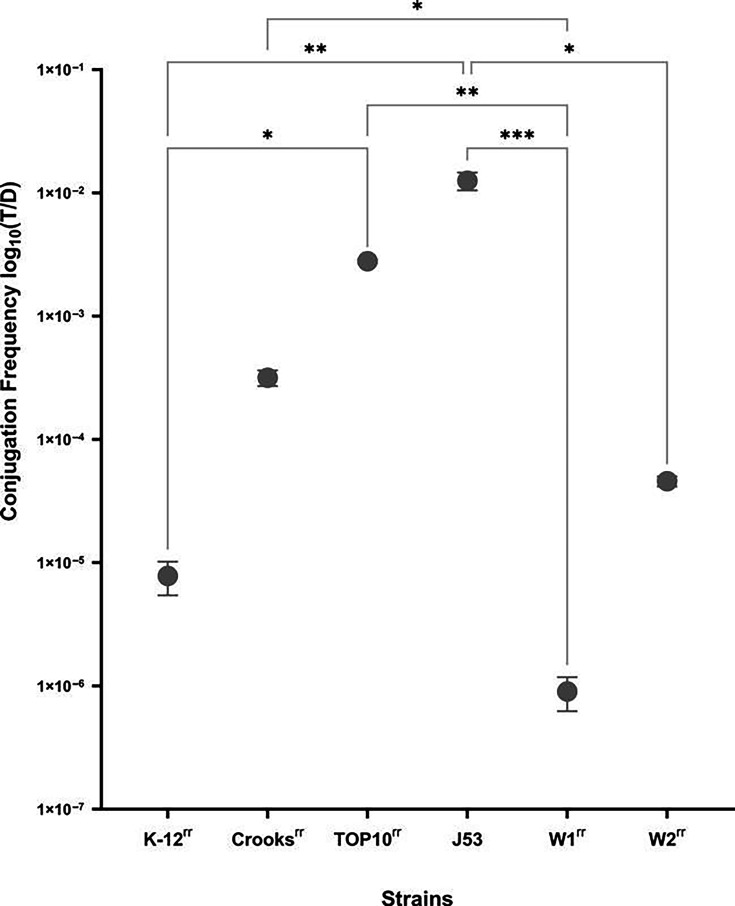
Conjugation frequencies of p201809181.3 from a wild *E. coli* donor strain to lab and wild *E. coli* recipient strains. Conjugation frequencies are given as the ratio of the number of transconjugants to the number of initial donor colonies. Points are means, and error bars are sd. Lines represent Kruskal–Wallis tests with an uncorrected Dunn’s multiple comparison post hoc test, and a 95% CI. Asterisks indicate comparisons with *P*≤0.05, in which *P* values are reported as GraphPad style: 0.1234 (ns), 0.0332 (*), 0.0021 (**), 0.0002 (***) and <0.0001 (****). Each conjugation experiment was quantified in triplicate.

For each assay, we revived the donor and the recipient strains by streaking them on MKL plates supplemented with the appropriate antibiotic: ceftriaxone (2 µg ml^−1^) for the donor and rifampicin (100 µg ml^−1^) for all recipients except for J53, which was grown with sodium azide (200 µg ml^−1^). Ceftriaxone is a third-generation cephalosporin to which the donor strain is resistant, because of the *bla*_CTX-M_ gene on p201809181.3 [27,33]. We eliminated antibiotic traces by resuspending one colony of each strain in 1 ml of 0.85% saline solution and then pelleting the cells by centrifugation at 3420 ***g*** for 30 min, after which we resuspended the pellet in 1 ml of fresh LB [34]. We mixed the donors and recipients at a 1:1 ratio to a final volume of 1 ml. We determined the initial cell titres of the donor and recipient strains using viable colony counts from plating 100 µl of a 10^−6^ dilution of this initial mixture on MKL agar+ceftriaxone (2 µg ml^−1^) for the donor and MKL agar+rifampicin (100 µg ml^−1^) for the recipients. We incubated the mixtures for 1 h at 37 °C without shaking [35], after which we quantified transconjugants by plating 10^0^, 10^−1^, 10^−2^, 10^−3^ or 10^−4^ dilutions onto MKL agar plates containing both antibiotics (ceftriaxone and rifampicin or sodium azide for J53 strain) and counting the resulting colonies after 24 h of incubation at 37 °C. We calculated conjugation frequencies as the ratio of the number of transconjugants (i.e. recipient cells that have received the plasmid) to the total number of donors (Figs 1 and S1, available in the online Supplementary Material) [36]. We performed all conjugation assays with threefold replication. To validate our findings, we conducted an additional experiment with *E. coli* ATCC 8739 strains modified for rifampicin and gentamicin resistance. The rifampicin-resistant strain, carrying the plasmid, was competed against the gentamicin-resistant, plasmid-free strain in LB medium under agitation (200 r.p.m.) for 24 h. Plating on MKL medium with rifampicin, gentamicin and ceftriaxone revealed no colonies, suggesting that conjugation events, if any, were below the detection threshold (<10 colonies) and did not influence the fitness results.

We isolated six transconjugants of each recipient strain and verified their AMR phenotypes (Table S1). We confirmed that the plasmid had successfully transferred to the recipient by PCR screening with plasmid-specific primers and then also verified the genotypes of the transconjugants by sequencing the *fumC* and *fliC* genes [30,37] (Tables S2 and S3). In all cases, we set up PCR reactions as follows: 12.5 µl of Q5 High Fidelity Master Mix, 1.25 µl of the corresponding primer, 9 µl of nuclease-free diH_2_O and 1 µl of DNA template. We submitted the *fumC* and *fliC* amplicons to MACROGEN (South Korea) for sequencing. We uploaded complete sequences to GenBank (accession numbers: PP827162, PP827163, PP827164 and PP827165 for *fumC* and PP823980, PP823981, PP823982 and PP823983 for *fliC*.)

### Competition assay

**Fig. 2.**
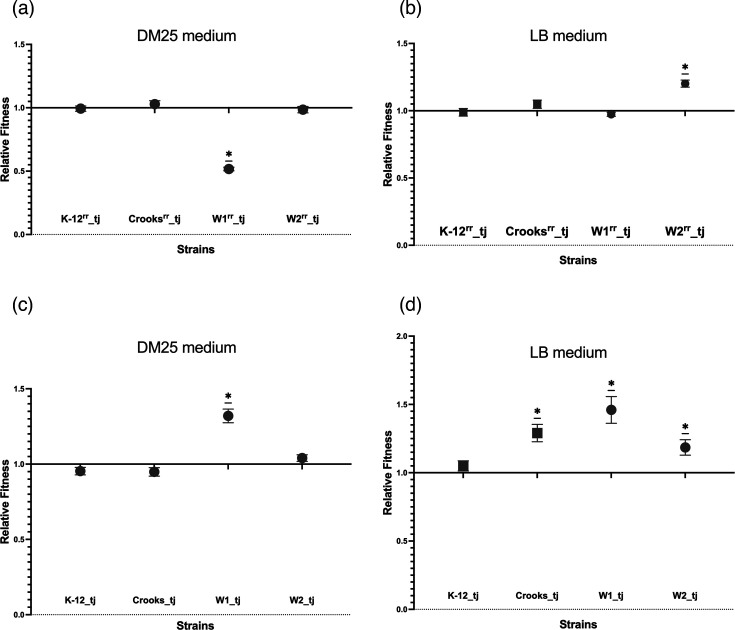
Relative fitness effects of p201809181.3 in laboratory and natural *E. coli* strains, as measured in DM25 (a, c) and LB media (b, d). *E. coli* rifampicin-resistant strains were tested in (**a)** and (**b)**, and parenteral rifampicin-sensitive strains were tested in (**c)** and (**d)**. The means and their sd are shown. Asterisks represent one-sample Wilcoxon test results with *P*≤0.05. *P* values are reported in GraphPad style: 0.1234 (ns), 0.0332 (*), 0.0021 (**), 0.0002 (***) and <0.0001(****). Each competition experiment was conducted with six replicates.

We assessed the fitness effects of p201809181.3 by competing each one of the plasmid-free strains against their corresponding transconjugants in pairwise, head-to-head competition assays in Davis–Mingioli (DM) medium supplemented with 25 mg ml^−1^ glucose (DM25) as described by Barrick *et al.* [38]. Briefly, we separately inoculated the competitor strains into LB and incubated them at 37 °C, with 200 r.p.m. orbital shaking speed for 24 h. We then diluted each culture 100-fold in sterile 0.85% saline and transferred 100 µl of the diluted cultures to 9.9 ml of fresh DM25. We incubated these cultures for 24 h under the same conditions as the LB cultures. This growth cycle in DM25 allowed the cultures to acclimate (precondition) to the medium and conditions in which competitions occurred. After 24 h, we transferred 50 µl of each pair of competitors to 9.9 ml of fresh DM25 with sixfold replication. We vortexed these co-cultures thoroughly and spread 100 µl of 10^2^ dilutions of each on both an unamended MKL plate and one supplemented with 2 µg ml^−1^ ceftriaxone. We then incubated the plates at 37 °C for 24 h, after which we counted the colonies that had arisen on each to provide the d0, initial enumerations of the two competitors. We incubated the competition cultures at 37 °C with 200 r.p.m. orbital shaking speed for 24 h, after which we spread 100 µl of 10 000-fold dilutions of each on MKL plates, again both unamended and amended with 2 µg ml^−1^ ceftriaxone to provide the d1, post-competition competitor enumerations. Transconjugant competitor titres were calculated using the mean colony counts from ceftriaxone-amended plates, while non-transconjugant competitor titres were enumerated by subtraction of the mean number of transconjugant colonies from the mean total number of colonies on the unamended plates. In the case of LB competitions, the same protocol was followed, save for the use of LB for the competition medium, instead of DM25 (Figs 2, S2 and S3).

We calculated the fitness of the transconjugants relative to their respective non-transconjugant competitors as described by Wiser and Lenski [39]: *w*=ln [Eplasmid 24 h/ Eplasmid 0 h]/ln [E 24 h/E 0 h], where *w* is the relative fitness, ‘Eplasmid 24 h’ is the number of transconjugant cells at 24 h, ‘Eplasmid 0 h’ is the number of transconjugant cells at 0 h, E 24 h is the number of non-transconjugant cells at 24 h and E 0 h is the number of non-transconjugant cells at 0 h.

To confirm the results of the competition experiments and rule out potential artefacts, we conducted two additional competitions between W1 strains (with and without the plasmid) and the Lac^−^ strain *E. coli* 14.SA.05 (Figs S4 and S5). These competitions were carried out as described above.

### Growth curve

We used spectrophotometric measures of bacterial growth to quantify the fitness effects of the rifampicin-resistant mutation we used as a marker in our conjugation experiments. Briefly, we streaked the parental strains W1 and W2 (with and without plasmid) onto MKL plates and incubated them overnight at 37 °C. We then started six independent cultures in LB medium and grew them overnight at 37 °C with 200 r.p.m. orbital shaking speed. We diluted the overnight cultures 1:100 into fresh LB medium. We also transferred 100 ml of this suspension to spectrophotometer cuvettes and incubated them at 37 °C with 200 r.p.m. orbital shaking speed for 12 h. We measured the change in OD at 600 nm every 30 min (Fig. S6). We determined the growth rates and other curve parameters using the GrowthCurver R package [40]. We defined the relative growth rate as the ratio of the plasmid-bearing cell growth rate to that of the plasmid-free cell (Table S4).

### Plasmid characterization

The p201809181.3 plasmid was originally sequenced, assembled and characterized by Salinas *et al.* [27]. In brief, plasmid DNA extracted from the donor strain was sequenced with the Rapid Barcoding Sequencing Kit (SQK-RBK004) (Oxford Nanopore Technologies) on a MinION Mk1B sequencing device using the MinKNOW software (Oxford Nanopore Technologies). *De novo* assembly with filtered reads obtained was performed through the Flye assembler 2.8.1-b1676 [41]. The circularized plasmid was annotated with the National Center for Biotechnology Information (NCBI) Prokaryotic Genome Annotation Pipeline (PGAP), and the AMR genes and plasmid type were identified using Resfinder [42] and PlasmidFinder [43], respectively, with ABRicate tool 1.0.1 [44]. We also carried out an independent annotation of the complete plasmid sequence using PATRIC, PROKKA and BAKTA (Table S5) to confirm these details. We used SnapGene Viewer to complete the annotations and create the plasmid gene map (Fig. S7).

### Statistical analysis

We compared the mean conjugation frequency between each strain using a nonparametric Kruskal–Wallis test and a post hoc Dunn’s multiple comparison test. We used a Mann–Whitney test to compare the relative fitness means obtained from our fitness assays conducted in DM and LB. We used a one-sample Wilcoxon test to analyse relative fitness using an expected mean of 1.0, which corresponds to a neutral fitness cost. All statistical analysis and graphing were carried out using GraphPad Prism 10.

## Results

### Conjugation frequency

We found that the two wild isolates had significantly lower conjugation frequencies than two of the laboratory strains: 9.021e−007±2.7e−007 for W1^rr^ and 4.592e−005±4.262e−006 for W2^rr^ versus 0.01257±0.002072 for J53^rr^ and 0.002797±0.0001104 for TOP10^rr^. By contrast, the wild isolates had conjugation frequencies that were significantly higher than the 7.827e−006+2.384e−006 rate observed for K-12^rr^ (*P*=0.0007).

### Fitness effects of p201809181.3 in laboratory and natural *E. coli* strains

Relative fitness varies depending on the context in which it is measured, including the culture conditions and the medium used. We chose to measure fitness in both rich medium, LB [20,45,47], and minimal medium, DM25 [48,50]. We made these choices because a minimal, nutrient-limited medium is often considered to be a more stressful environment, while a rich medium is usually considered to provide a more relaxed one [51]. Thus, measuring fitness in both media types would provide a better sense of the plasmid’s fitness effects across different conditions. Surprisingly, one-sample Wilcoxon tests showed that p201809181.3 had no significant fitness effect on most strains in either competition medium (Fig. 2). The sole exception in both media was W1^rr^. In DM25, carriage of p201809181.3 caused this strain a ~48% loss of fitness in DM25 (*w*=0.52±0.01, *P*=0.0312) (Fig. 2a), while the plasmid actually improved its fitness by ~20% in LB (*P*=0.0312) (Fig. 2b)

We were unable to assess the fitness effects of p201809181.3 for J53 and TOP10^rr^ in DM25, as neither was able to in minimal medium because J53 is a leucine and proline auxotroph and TOP10^rr^ cannot use glucose. However, in LB, the plasmid had insignificant effects on the fitness of both strains (Fig. S1).

We hypothesized that the lack of fitness cost associated with the plasmid might stem from interactions between it and the mutation that conferred rifampicin resistance upon the recipient strains. To test this hypothesis, we conjugated the plasmid into an *E. coli* strain (TOP10) with a Lac^−^ marker phenotype to differentiate it from the donor strain. We then used the resulting transconjugant as a donor with which to conjugate the plasmid into K-12, Crooks, W1 and W2. We conducted competition assays to measure the plasmid’s fitness effects in these strains. Remarkably, we found that the plasmid conferred small fitness benefits in both rifampicin-sensitive genetic backgrounds, as measured in DM25. Relative to their respective plasmid-free competitors, W1(p201809181.3) had a fitness of 1.32±0.05 and W2(p201809181.3) had a fitness of 1.04±0.02. One-sample Wilcoxon tests showed that the fitness effect was significant in W1 (*P*=0.0312), but insignificant in W2 (*P*=0.1875). In competitions carried out in LB, the presence of the plasmid conferred statistically significant fitness improvements in three transconjugants: ~29% (*w*=1.29±0.04, *P*=0.0312) for Crooks, ~46% (*w*=1.46±0.10, *P*=0.0312) for W1 and ~19% (*w*=1.19±0.06, *P*=0.0312) for W2 (Figs 2d and S3). We also observed a slight but statistically insignificant fitness improvement of ~5% (*w*=1.05±0.04, *P*=0.2500) for K-12 in LB.

### Excluding artefactual effects from additional genes encoded by p201809181.3

Given our prior results, we sought to confirm that p201809181.3 confers improved fitness with a follow-up experiment in which we competed W1 with and without the plasmid against *E. coli* strain 14.SA.05, a wild isolate that is unable to ferment lactose, which provided a means of differentiating the competitors on MKL agar. This experiment was specifically designed to rule out the possibility that the observed higher fitness is, in fact, an artefactual result of conjugation. While the 14.SA.05 exhibited higher fitness than W1 or its transconjugant, the latter displayed higher relative fitness than the former (Fig. S5). This finding was supported by growth curve analyses of rifampicin-sensitive and rifampicin-resistant W1 strains and their respective transconjugants (Fig. S6). We also compared the numbers of lactose-negative colonies co-cultured with the W1-derived transconjugants to rule out any antagonistic effects between the plasmid and the rifampicin-resistant mutation.

## Discussion

Plasmids carrying antibiotic-resistant genes, such as ESBLs, can impose a fitness cost on their hosts owing to the metabolic burden of maintaining and replicating the plasmid and expressing its encoded genes. This burden can cause slower growth rates, reduced competitiveness and altered physiological functions compared to plasmid-free strains [24]. However, these costs to the host bacteria can sometimes be mitigated by the accumulation of compensatory mutations or by the evolution of mechanisms that enhance plasmid stability and function [52,53]. The *bla*_CTX-M-55_ gene is a member of the CTX-M family of ESBLs, which encode *β*-lactamases that confer host resistance to *β*-lactam antibiotics like penicillins and cephalosporins. Like other ESBLs, *bla*_CTX-M-55_ is commonly spread by plasmids, including p201809181.3, a multireplicon plasmid (IncFII:IncN) with various post-segregational killing systems, including toxin-antitoxin, anti-restriction, methylation and partition control systems (Fig. S7 and Table S5). It also carries a cluster of resistance genes, which include *bla*_TEM_ and *fosA3* in addition to *bla*_CTX-M-55_ [27]. Like many plasmids, p201809181.3 is able to persist in host cells even without direct antibiotic selection to maintain it. We have described our exploration of the impact of maintaining this large plasmid on *E. coli* strains in the absence of antibiotics with the intention of providing insights into how such plasmids are maintained in bacterial communities despite their inherent costs under relaxed selection.

The ability of some plasmids to disseminate to other hosts in the intestine through conjugation has been proposed to explain their maintenance in the population [20,54]. However, we found that p201809181.3 displays higher conjugation frequencies in two of the laboratory strains we studied, J53 and TOP10, than it does in natural commensal strains. However, the plasmid also shows relatively lower conjugation rates in K-12^rr^ than in the other laboratory strains. This disparity might be owing to J53 and TOP10 having had a longer evolutionary history in the laboratory than K-12 [55,59]; however, further experiments are needed to validate this assumption. Our observations would be consistent with this possibility, as an adaptation to laboratory conditions may select for compromised defences against plasmids [56,57, 59, 60]. Moreover, unlike the other laboratory strains, *E. coli* K-12 has genomic features that could potentially interfere with p201809181.3’s compatibility, including a lambda prophage and integrated F plasmid [55,58, 61]. In general, plasmid p201809181.3 transfers at lower rates among WT *E. coli* than laboratory strains, which is contrary to the proposed explanation for its maintenance, but which has also been described previously [36,52].

It has been postulated that low plasmid conjugation rates may be compensated for by low fitness costs [20]. Indeed, contrary to theoretical expectations that plasmids should carry large fitness costs for their hosts, we found that p201809181.3 carries no fitness costs in either wild commensal or laboratory *E. coli* strains. The sole exception was the wild strain, W1, though only its rifampicin-resistant derivative (W1^rr^) exhibited reduced fitness associated with the plasmid and, even then, only in a minimal medium. Low fitness costs of plasmids carrying AMR have been described before [45,62,65].

As we had expected, we found that the fitness effects of p201809.181.3 on host bacteria varied with both the host strain and the competition medium used (DM25 or LB). This variation may explain inconsistencies in the fitness effects that have been reported for plasmids in the literature [20,45,47, 63, 66]. Some have suggested that plasmids may tend to exert lower fitness burdens when measured in a rich medium than minimal medium because of the stress that the latter places on cells [51]. We therefore suspect that a minimal medium may allow fitness measures that are closer to those bacteria experience in the intestine, where they are under intense competition for nutrients with other members of the resident microbiota [67]. Our finding that p201809181.3 does not incur a fitness cost in DM25, while enhancing fitness by ~25% in LB medium, is in line with these expectations (Fig. S10 A). Our findings are also striking because they contradict previous studies that have found that large plasmids like p201809181.3 (99774 bp) tend to carry high fitness costs [68].

We initially performed our competition assays with transconjugants of rifampicin-resistant mutants of our strain collection. However, we found clear differences in the fitness effects of the plasmid between the Rif^r^ mutants and their parent clones (Figs S8, S9 and S10). These results suggest that there are negative epistatic interactions between the plasmid and rifampicin-resistant mutations in the RNA polymerase beta subunit gene [69]. Mutations that confer rifampicin resistance, particularly those in the *rpoB* gene that affect RNA polymerase function, can disrupt the balance of gene expression and cellular metabolism [70]. These resistance mutations may also compromise plasmid stability and functionality, likely due to altered metabolic and stress response pathways that reduce plasmid fitness [71,72]. As a result, plasmids may become less stable and less effective in expressing their encoded traits. Thus, rifampicin-resistant mutations can pose challenges for plasmid maintenance and function, demonstrating a complex interplay in which resistance and plasmid carriage can be mutually detrimental [73,74]. Our findings differed from those of a study that investigated the fitness costs associated with 13 plasmids carrying antibiotic-resistant genes in *E. coli* strains resistant to nitrofurantoin, ciprofloxacin and streptomycin, which showed significant fitness costs in 9 out of the 13 plasmids when tested in the sensitive parental strain. However, none of the plasmids examined adversely affected the growth rates of the antibiotic-resistant descendant strains [75].

We sought to rule out any potential artefactual effects caused by other additional genes encoded by p201809181.3 by performing competitions between W1 with and without the plasmid and a Lac^−^ WT *E. coli* competitor. This competitor exhibited greater fitness compared to the W1 strains, both with and without the plasmid, though the transconjugant with p201809181.3 showed higher fitness than its plasmid-free counterpart. Analysis of growth curves and relative growth rates verified these findings.

How might p201809181.3 confer increased fitness? Plasmids often carry genes that code for novel metabolic traits and virulence genes, which can potentially improve host fitness [76]. Plasmids can have direct, complex interactions that influence host metabolism [53,77,79]. Moreover, an analysis of over 1000 plasmid-borne genes identified a large number of genes involved in cellular metabolism and stress response, which occurred at frequencies comparable to those of antibiotic-resistant genes [79]. A study also demonstrated that various plasmids encoding resistance to a range of antibiotics in *E. coli* K-12 MG1655 can reprogramme the expression of metabolic genes, with effects that may depend on the genetic background of the host and the specific plasmid [78]. Our plasmid carries the *proQ/finO* gene, which has been reported to lower the fitness costs of IncI2 plasmids by reducing the plasmid’s copy number to one per cell [80]. We also identified genes belonging to an operon coding for an arsenic pump (*ydF*, *ydeA* and *arsR*), as well as a negative regulator (ANR), a lipoprotein, four hypothetical proteins, a glutathione synthetase and a metalloprotein, the last two of which are flanked by two copies of insertion sequence IS26, as well as the resistance genes. ANR belongs to a recently identified, large family of small regulatory proteins present in multiple enteric bacterial pathogens, which have been proposed as transcriptional regulators associated with fitness and virulence [81]. It is unclear if these genes might be responsible for the fitness improvements we observed, or how they might be manifested. Follow-up work on this question may involve moving these genes to host chromosomes to directly test if they might be responsible for the fitness effects we identified. Directed mutagenesis of these genes on p201809181.3 could also be helpful in answering this question, as would be metabolic and transcriptomic analysis.

Our findings highlight the complex ways in which plasmids may affect the fitness of other hosts and suggest that the prevalence and persistence of plasmids in bacterial populations, even under relaxed selection, are not as paradoxical as often thought. Indeed, if, as we have shown with p201809181.3, plasmids confer more general adaptive benefits beyond AMR to their hosts, then they would be expected to be maintained in the absence of antimicrobials. Further study of these advantages and their origins is warranted, as they might interact synergistically with AMR genes in ways that increase rates of AMR spread. Indeed, improving our understanding of how plasmids affect host fitness and how the genes they carry might interact in producing these effects will be vital for developing strategies to combat the spread of resistance in microbial populations. Improved understanding is particularly needed with regard to plasmids that carry *bla*_CTX-M_ genes, given their growing role in AMR.

## supplementary material

10.1099/mic.0.001525 Uncited Supplementary Material 1.

## Abbreviations

AMR: antimicrobial resistance
DM: Davis–Mingioli
ESBL: extended-spectrum *β*-lactamase
ESC: expanded-spectrum cephalosporin
LB: lysogeny broth
MKL: MacConkey lactose
