# Supplementary material for: A plasmid with the blaCTX-M gene enhances the fitness of Escherichia coli strains under laboratory conditions
Source: Microbiology (Reading). 2025 Jan 30;171(1):001525. doi: 10.1099/mic.0.001525 (PMC11781320; doi:10.1099/mic.0.001525)
Supplement: Uncited Supplementary Material 1. [file mic-171-01525-s001.pdf]

Supplementary Materials

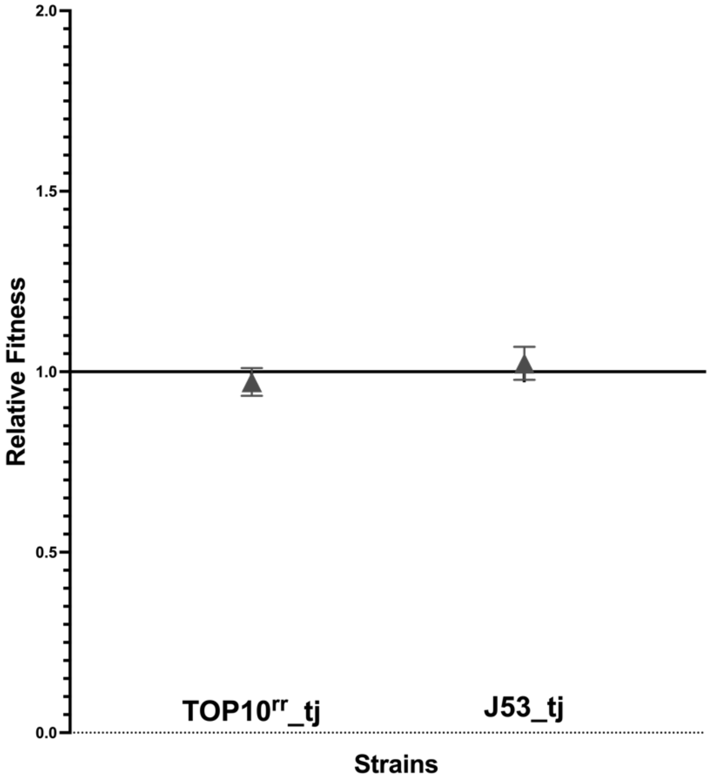

Supplementary Figure 1. Relative fitness cost of carriage a *bla*$_{CTX-M}$ 55 plasmid for TOP10^{rr} and J53 engineered *E.coli* strains in LB medium. Asterisks represented one sample Wilcoxon test results with p values less than or equal to 0.05. Each competition experiment was conducted with six replicates.

Supplementary Table 1. Antimicrobial susceptibility profiles by disc diffusion method according to Clinical and Laboratory Standards Institute (CLSI) guidelines.

Strain	PENICILLINS	FOSFOMYCINS	AMINOGLYCOSIDES			QUINOLONES AND FLOUROQUINOLONES	PHENICOLS	FOLATE PATHWAY ANTAGONISTS	TETRACYCLINES	MACROLIDES	CEPHEMS					CARBAPENEMS	NITROFURANS
	Ampicillin	Fosfomycin	Streptomycin	Gentamicin	Kanamycin	Ciprofloxacin	Chloramphenicol	Trimethropim-Sulfamethoxazole	Tetracycline	Azithromycin	Cefazolin	Cefuroxime	Ceftazidime	Ceftriaxone	Cefepime	Imipenem	Nitrofurantoin
Donor	R	R	I	S	I	R	R	R	R	S	R	R	S	R	SDD	S	S
K-12^{rr}	S	S	S	S	S	S	S	S	S	S	S	S	S	S	S	S	S
K-12^{rr}_tj	R	R	S	S	S	S	S	S	S	S	R	R	S	R	SDD	S	S
Crooks^{rr}	S	S	S	S	S	S	S	S	S	S	S	S	S	S	S	S	S
Crooks^{rr}_tj	R	R	S	S	S	S	S	S	S	S	R	R	S	R	SDD	S	S
TOP10^{rr}	S	S	R	S	S	S	S	S	S	S	S	S	S	S	S	S	S
TOP10^{rr}_tj	R	R	R	S	S	S	S	S	S	S	R	R	S	R	S	S	S
J53	S	S	S	S	S	S	S	S	S	S	S	S	S	S	S	S	S
J53_tj	R	R	S	S	S	S	S	S	S	S	R	R	S	R	SDD	S	S
W1^{rr}	S	S	S	S	S	S	S	S	S	S	S	S	S	S	S	S	S
W1^{rr}_tj	R	R	S	S	S	S	S	S	S	S	R	R	I	R	SDD	S	S
W2^{rr}	S	S	S	S	S	S	S	S	S	S	S	S	S	S	S	S	S
W2^{rr}_tj	R	R	S	S	S	S	S	S	S	S	R	R	S	R	SDD	S	S
K-12	S	S	S	S	S	S	S	S	S	S	S	S	S	S	S	S	S
K-12_tj	R	R	S	S	S	S	S	S	S	S	R	R	S	R	SDD	S	S
Crooks	S	S	S	S	S	S	S	S	S	S	S	S	S	S	S	S	S
Crooks_tj	R	R	S	S	S	S	S	S	S	S	R	R	S	R	SDD	S	S
W1	S	S	S	S	S	S	S	S	S	S	S	S	S	S	S	S	S
W1_tj	R	R	S	S	S	S	S	S	S	S	R	R	I	R	SDD	S	S
W2	S	S	S	S	S	S	S	S	S	S	S	S	S	S	S	S	S
W2_tj	R	R	S	S	S	S	S	S	S	S	R	R	S	R	SDD	S	S

Supplementary Table 2. Primers used for identification of the strain and presence of the study plasmid.

Gene	Primer sequence (5' to 3')		Annealing Temp
	Forward	Reverse	
fumC	TCACAGGTCGCCAGCGCTTC	GTACGCAGCGAAAAAGATTC	65°C
fliC	GGTCAGGCGATTGCTAACCG	GACACTTCGGTCGCGTAGTC	60°C
**IncFII**	GTCTTGAACTTTTCCGGGCA	TGTGGATGTACAGGAGCCAG	67°C
**IncN**	GAGATGGCTGTACTCGGTGA	CCCAGATACCACGAGCTGAT	67°C
bla_{CTX-M}	ATGTGCAGYACCAGTAARGTKATGGC	TGGGTRAARTARGTSACCAGAAYCAGCGG	65°C

Supplementary Table 3. Genotypic profile of donor, recipients and transconjugants strains.

Strain	Strain ID		Study Plasmid		
	fumC	*fliC*	IncFII	IncN	bla_{CTX-M}
Donor	11	38	+	+	+
K-12	11	48_1	+	-	-
K-12_tj	11	48_1	+	+	+
Crooks	7	20	-	-	-
Crooks_tj	7	20	+	+	+
TOP10	11	48_2	-	-	-
TOP10_tj	11	48_2	+	+	
J53	11	48_1	-	-	-
J53_tj	11	48_1	+	+	+
W1	4	12	-	-	-
W1_tj	4	12	+	+	+
W2	4	8	-	-	-
W2_tj	4	8	+	+	+

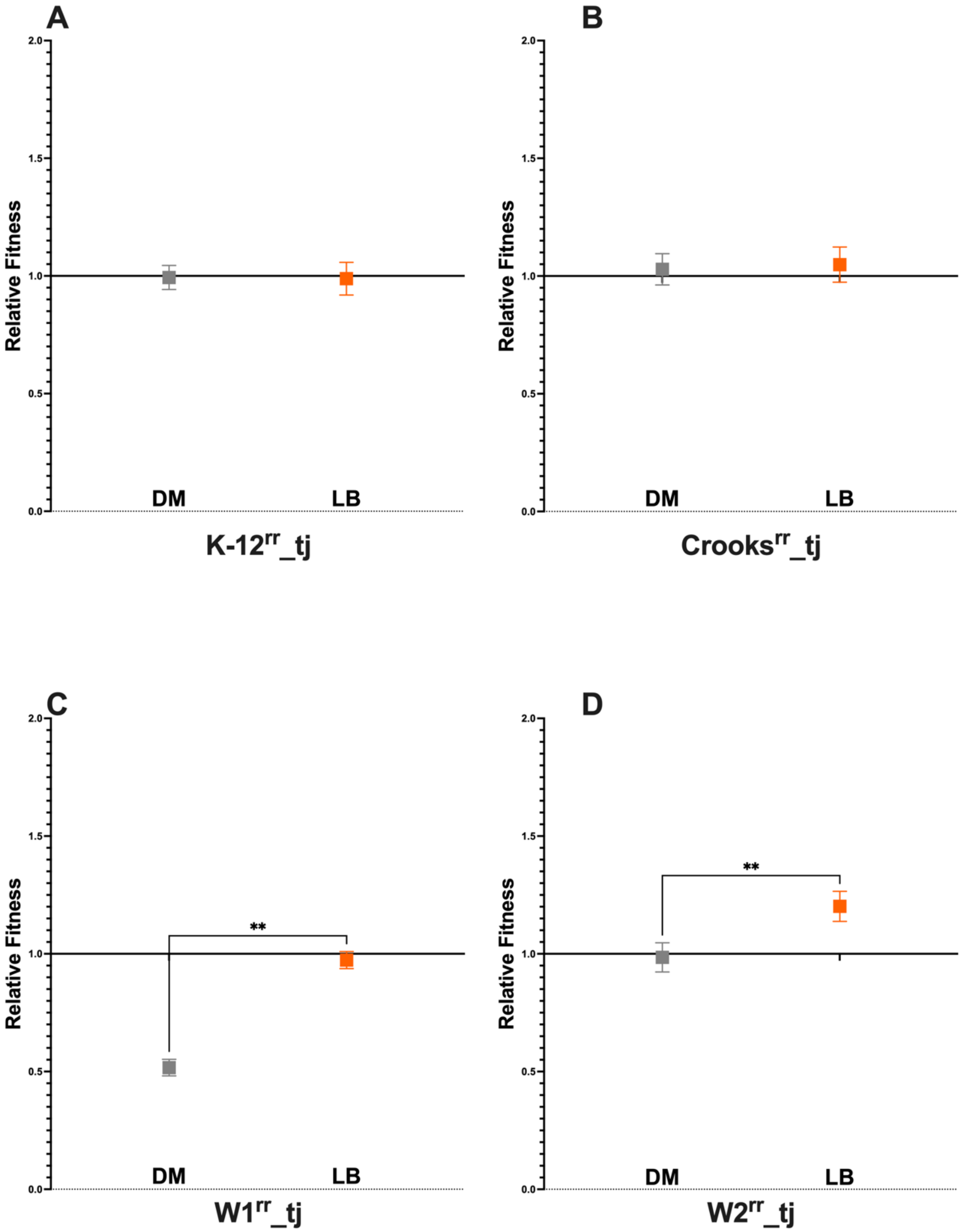

Supplementary Figure 2. Relative fitness assayed in DM versus LB media from rifampicin resistant strains able to grow in both media. **A** correspond to the relative fitness of K-12^{rr} strain in DM (gray) and in LB (orange), **B** to the relative fitness of Crooks^{rr} strain in DM (gray) and in LB (orange), **C** to the relative fitness of W1^{rr} strain in DM (gray) and in LB (orange), **D** to the relative fitness of W2^{rr} strain in DM (gray) and in LB (orange). Asterisks and lines represented Mann-Whitney test results with p values less than or equal to 0.05 for the comparison of each strain in each medium.

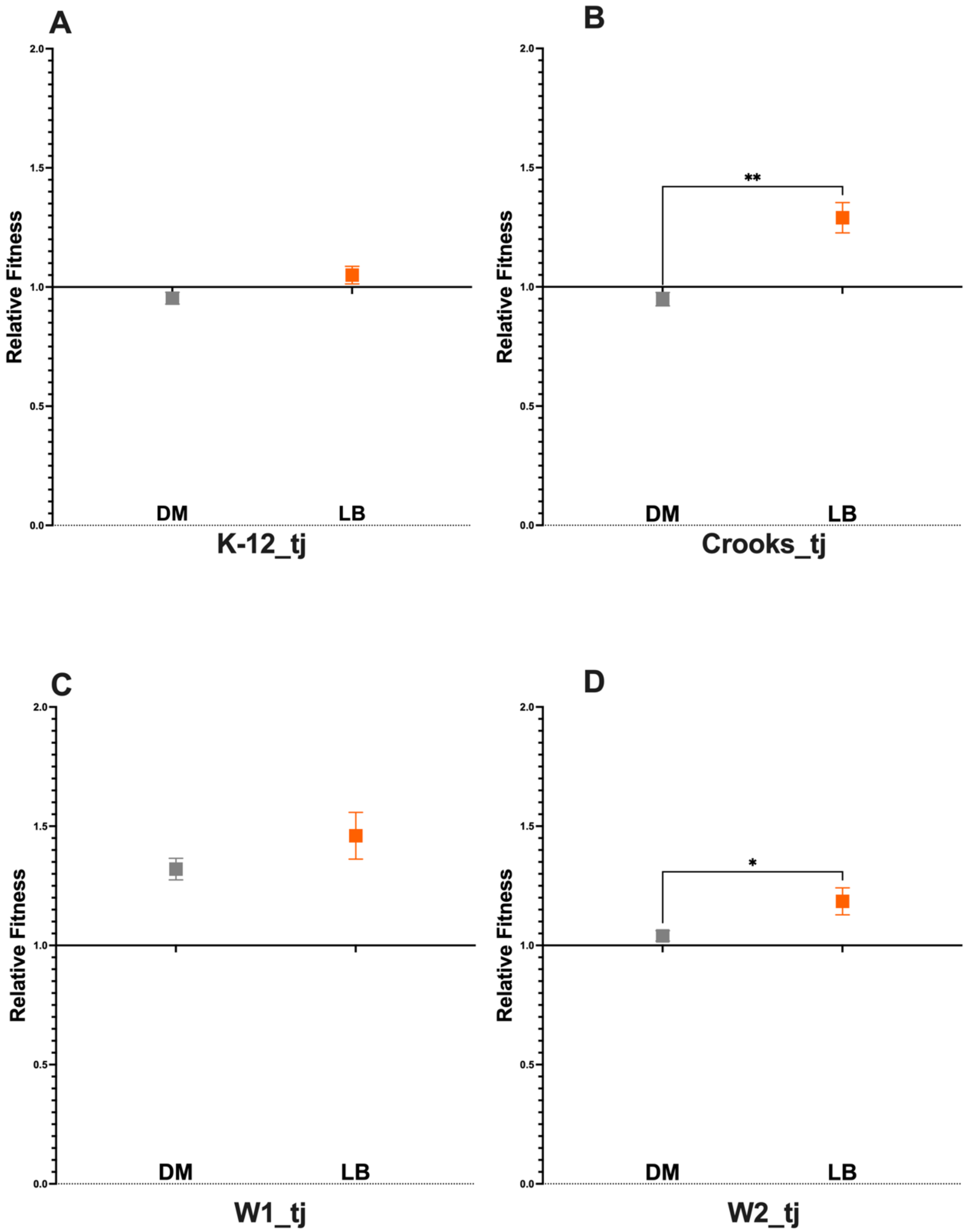

Supplementary Figure 3. Relative fitness assayed in DM versus LB media from original sensitive strains able to grow in both media. **A** correspond to the relative fitness of K-12 strain in D (gray) and in LB (orange), **B** to the relative fitness of Crooks strain in DM (gray) and in LB (orange), **C** to the relative fitness of W1 strain in DM (gray) and in LB (orange), **D** to the relative fitness of W2 strain in DM (gray) and in LB (orange). Asterisks and lines represented Mann-Whitney test results with p values less than or equal to 0.05 for the comparison of each strain in each medium.

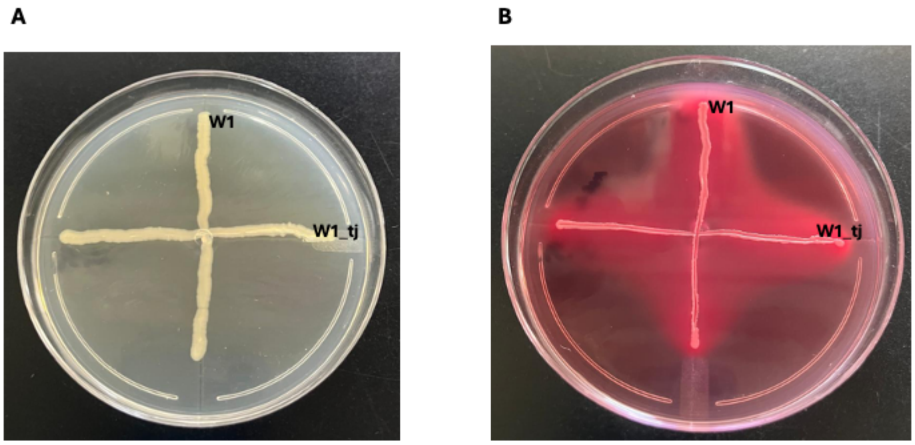

Supplementary Figure 4. Growth of parenteral strain without plasmid versus parenteral strain with plasmid, to verify possible antagonistic relationships. **A**: tested on LB agar, and **B**: tested on MKL agar. Each experiment was conducted in triplicate.

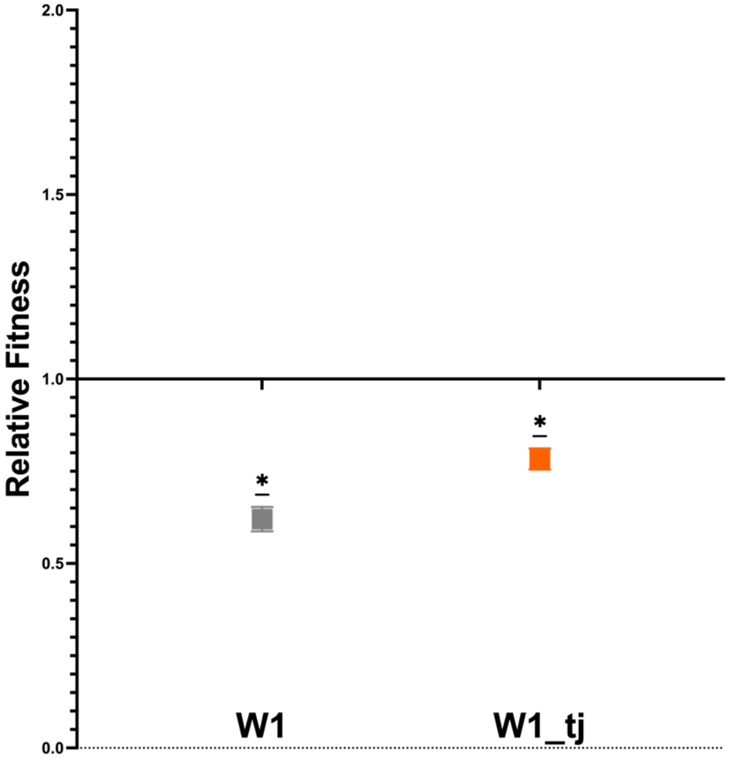

Supplementary Figure 5. Relative fitness of W1 parenteral strain in DM against a lactose negative common competitor. Asterisks represented one sample Wilcoxon test results with $p \leq 0.05$. Each competition experiment was conducted with six replicates.

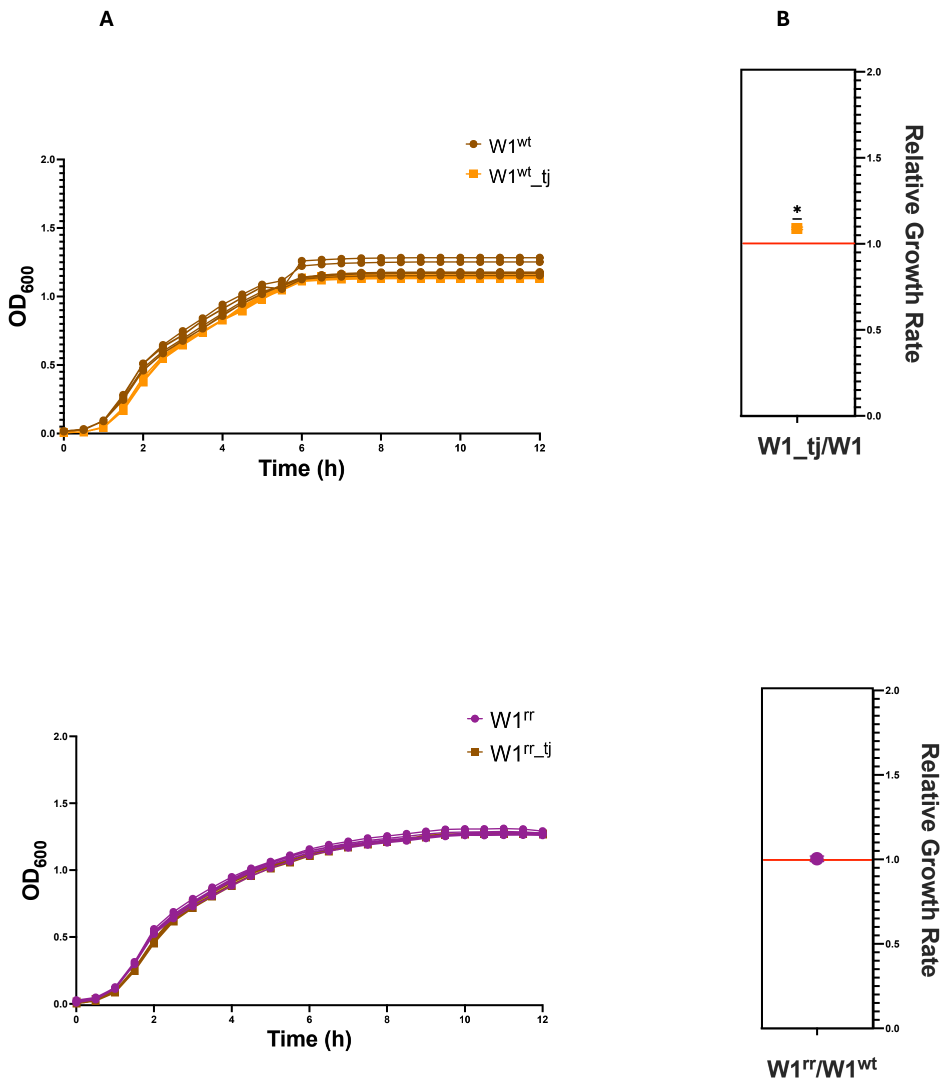

Supplementary Figure 6. Growth curve (**A**) and relative growth rate (**B**) of the different W1 variant strains. Growth curves were quantified for six replicates per strain.

Supplementary Table 4. Growth curve parameters of the different W1 variant strains curves.

Strain	growth rate (h^{-1})	generation time (min)	lag time (h)	carrying capacity (OD_{600})
**W1^{rr}**	1,96	21,17	1,90	0,871
**W1^{rr}_tj**	1,96	21,24	2,00	0,871
**W1**	1,91	21,74	2,00	0,863
**W1_tj**	2,09	19,94	2,13	0,800

Supplementary Table 5. List of annotated p201809181.3 plasmid genes

Type	Start	Stop	Strand	Gene	Product
cds	1	686	+	repB	RepB family plasmid replication initiator protein
oriC	687	2200	?	oriV	origin of replication
cds	2258	2853	-		recombinase family protein
cds	2911	3136	-		transposase
cds	3074	3877	+	MobA/MobL	Mobilization protein
cds	3890	4473	+	MobX	Mobilization protein
cds	4516	5328	-		Helicase ATP-binding domain-containing protein
cds	5271	7158	-		DEAD/DEAH box helicase family protein
cds	7155	9202	-		site-specific DNA-methyltransferase (adenine-specific)
cds	9451	9654	-		HTH OST-type domain-containing protein (RNA nuclease)
cds	9697	9927	-		DUF3085 domain-containing protein
cds	9984	10103	-		(pseudo) hypothetical protein
cds	10175	10717	-		UDG domain-containing protein
cds	10701	11150	-		Periplasmic protein
cds	11161	11700	-		Lipoprotein
cds	11916	12062	-		Restriction endonuclease subunit S
cds	12220	12795	-		hypothetical protein
cds	12803	13081	+	repB	RepB family plasmid replication initiator protein
cds	13106	13789	+	tnp	IS26 (IS6 family IS15DII transposase)
cds	13857	14075	+	repB	(pseudo) RepB family plasmid replication initiator protein
cds	14272	14751	-		hypothetical protein
oriC	14462	15228	?	oriV	origin of replication

cds	15129	16564	-		glutathione synthetase
cds	16831	17534	+	tnp	IS26 (IS6 family IS15 transposase)
cds	17568	18016	-	fosA	FosA3/FosA4 family fosfomycin resistance (glutathione transferase)
cds	18401	19104	+	tnp	IS26 (IS6 family IS15DII transposase)
cds	19154	19896	+	blaTEM-1	broad-spectrum class A beta-lactamase TEM-1
cds	20152	20661	-		Metalloprotein/Tryptophan synthase (Secreted protein)
cds	20675	21549	-	blaCTX-M 55	CTX-M family extended-spectrum class A beta-lactamase
cds	21971	22675	-	tnp	IS26 (IS6 family IS15 transposase)
cds	23256	23939	+	tnp	IS26 (IS6 family IS15 transposase)
cds	24453	24713	+		DUF667 domain-containing protein
cds	25177	25413	-	ycdA	YcdA (swarming motility)
cds	25442	25723	+		Uncharacterized protein
cds	25860	26198	-	stbB	Plasmid segregation protein
cds	26199	27158	-	stbA	Plasmid segregation protein
cds	27672	27872	+		DNA methylase
cds	27872	28354	+		Methyltransferase
cds	28411	28575	+		Cytoplasmic protein
cds	28589	29023	+		DUF1380 domain-containing protein
cds	29023	29298	+		Transposase
cds	29376	29849	+	ychA	YchA
cds	29824	30300	+		Antirestriction protein
cds	30266	30691	+	yubI	Putative antirestriction protein YubI
cds	30738	31160	+		DUF1380 domain-containing protein
cds	31157	31348	+		Plasmid protein
cds	31661	33326	-	ltrA	group II intron reverse transcriptase/maturase
cds	33961	34461	-		Secreted protein
ncRNA	34627	34728	-	AS-pc01	Antisense to pHK01_035
cds	34723	34953	+	ydaB	YdaB
cds	35005	35373	+		DUF3560 domain-containing protein
cds	35902	36195	+		Lipoprotein
cds	36192	36362	+		Transposase
cds	36407	36892	+		MTS domain-containing protein
cds	36968	37132	+		hypothetical protein
cds	37115	37456	-		hypothetical protein
cds	37686	38325	+		Single-stranded DNA-binding protein
cds	38383	38616	+	ykfF	DUF905 domain-containing protein
cds	38677	40699	+	parB	ParB family partition protein
cds	40768	41202	+	psiB	conjugation system SOS inhibitor PsiB
cds	41199	41918	+	psiA	Protein PsiA
cds	41939	42154	-	sok	Protein sok (toxin)

ncRNA	42024	42200	+	sok	sok antitoxin (CssrC)
cds	42140	42289	+		Mok (plasmid maintenance)
cds	42232	42356	+		type I toxin-antitoxin system Hok (toxin)
cds	42674	42969	-	yehA	YehA
sorf	43157	43243	+		Single-stranded DNA-binding protein
cds	43267	43563	+	yeiA	YeiA
cds	43673	44494	+		DUF945 domain-containing protein
cds	44791	45438	-	yubQ	X polypeptide
oriT	45179	45648	?		origin of transfer
cds	45714	46097	+	traM	Relaxosome protein TraM
ncRNA-region	46185	46293	+		traJ 5' UTR
ncRNA	46218	46294	-	finP	FinP
cds	46288	46973	+	traJ	TraJ
cds	47408	47692	+	traA	type IV conjugative transfer system pilin TraA
cds	47707	48018	+	traL	type IV conjugative transfer system protein TraL
cds	48040	48648	+	traE	type IV conjugative transfer system protein TraE
cds	48648	49319	+	traK	type-F conjugative transfer system secretin TraK
cds	49319	50745	+	traB	Conjugal transfer protein TraB
cds	50735	51325	+	traP	Protein TraP
cds	51312	51509	+	trbD	Conjugal transfer protein TrbD
cds	51768	52283	+	traV	type IV conjugative transfer system lipoprotein TraV
cds	52418	52639	+	traR	conjugal transfer protein TraR
cds	52799	55425	+	traC	type IV secretion system protein TraC
cds	55422	55808	+	trbI	type-F conjugative transfer system protein TrbI
cds	55805	56437	+	traW	type-F conjugative transfer system protein TraW
cds	56434	57426	+	traU	Protein TraU
cds	57453	58345	+		Conjugative transfer protein
cds	58372	59010	+	trbC	type-F conjugative transfer system pilin assembly protein TrbC
cds	59007	59378	+		Transposase
cds	59404	59499	+		ATP-binding protein
cds	59499	59840	+		Transposase
cds	59840	60175	+	tnpA	IS66-like element accessory protein TnpA
cds	60175	60522	+	tnpB	IS66 family insertion sequence element accessory protein TnpB
cds	60542	62113	+	tnp	IS66 family ISCro1 transposase
cds	59499	62113	+		IS66
cds	62146	62532	+		Putative HNH endonuclease
cds	62593	64379	+	traN	type-F conjugative transfer system mating-pair stabilization protein TraN
cds	64402	64662	+	trbE	Conjugal transfer protein TrbE
cds	64807	65396	+	traF	type-F conjugative transfer system pilin assembly protein TraF

cds	65557	65792	+	traQ	Type-F conjugative transfer system pilin chaperone family protein
cds	65779	66312	+	trbB	Conjugal transfer protein TrbB
cds	66313	66662	+	trbJ	Conjugal transfer protein TrbJ
cds	66581	66973	+	trbF	Conjugal transfer protein TrbF
cds	66960	68332	+	traH	Conjugal transfer pilus assembly protein TraH
cds	68329	70065	+	traG	conjugal transfer mating pair stabilization protein TraG
ncRNA	69048	69133	-	AS-traG	Antisense to traG
cds	70062	71144	+	traG	Conjugal transfer protein TraG
cds	71177	71505	+	traS	(pseudo) protein traS
cds	71527	72261	+	traT	TraT complement resistance protein
cds	72398	73103	+	yhfA	YhfA
cds	73249	75507	+	traD	type IV conjugative transfer system coupling protein TraD
ncRNA	78351	78427	-	AS-traI	Antisense to traI
cds	75507	80770	+	traI	Protein TraI
cds	80790	81593	+	traX	conjugal transfer pilus acetylase TraX
cds	81592	82147	+	FinO	Fertility inhibition protein
cds	82277	82477	+		ANR family transcriptional regulator
	82520	82887	+	repA	RepA (N-domain)
cds	82859	83458	+		PIN7 domain-containing protein
cds	83520	83876	+		Rep-A-N domain-containing protein
ncRNA	84009	84166	+	sok	sok antitoxin (CssrC)
cds	84154	84303	+		Hok/gef toxin (pndA)
cds	84587	84835	+	copB	Protein CopB
ncRNA	84973	85062	-	copA	CopA-like RNA
sorf	85080	85154	+	tap	RepA leader peptide Tap
cds	85147	86019	+	repA	incFII family plasmid replication initiator RepA
oriC	86004	86365	?	oriV	origin of replication
cds	86321	86623	-		Transmembrane protein
cds	86942	87595	+		Intramembrane metalloprotease(microcin M activity)
cds	87687	87944	+	pemI	type II toxin-antitoxin system antitoxin PemI
cds	88151	88726	-	tnp	IS91 family IS1294 transposase
cds	88852	89349	-		Mobile element protein
cds	89359	89547	-		IS1294 transposase
cds	88151	89547	-		IS91
cds	89636	89731	-		hypothetical protein
cds	89721	90011	-		ATP-binding protein
cds	90305	90535	+	vapB	Vap B antotoxin

cds	90532	90948	+	vapC	VapC toxin
cds	91110	93083	-		ATPase (AAA-13 domain-containing protein)
cds	93127	93471	+		hypothetical protein
cds	93601	93858	+	ydfA	YdfA (arsenical pump membrane protein)
cds	93858	94448	+	ydeA	YdeA protein
cds	94694	96263	+		AAA family ATPase
cds	96454	97069	+	ProQ/FINO	ProQ/FINO
cds	96870	97112	-		Glycosyltransferase
cds	97362	98398	-		permease
cds	98503	98826	+	arsR	ArsR (Arsenical operon transcriptional repressor)
cds	99024	99727	+	tnp	IS6 family IS15 transposase
cds	99663	99773	-		Glutathione dehydrogenase

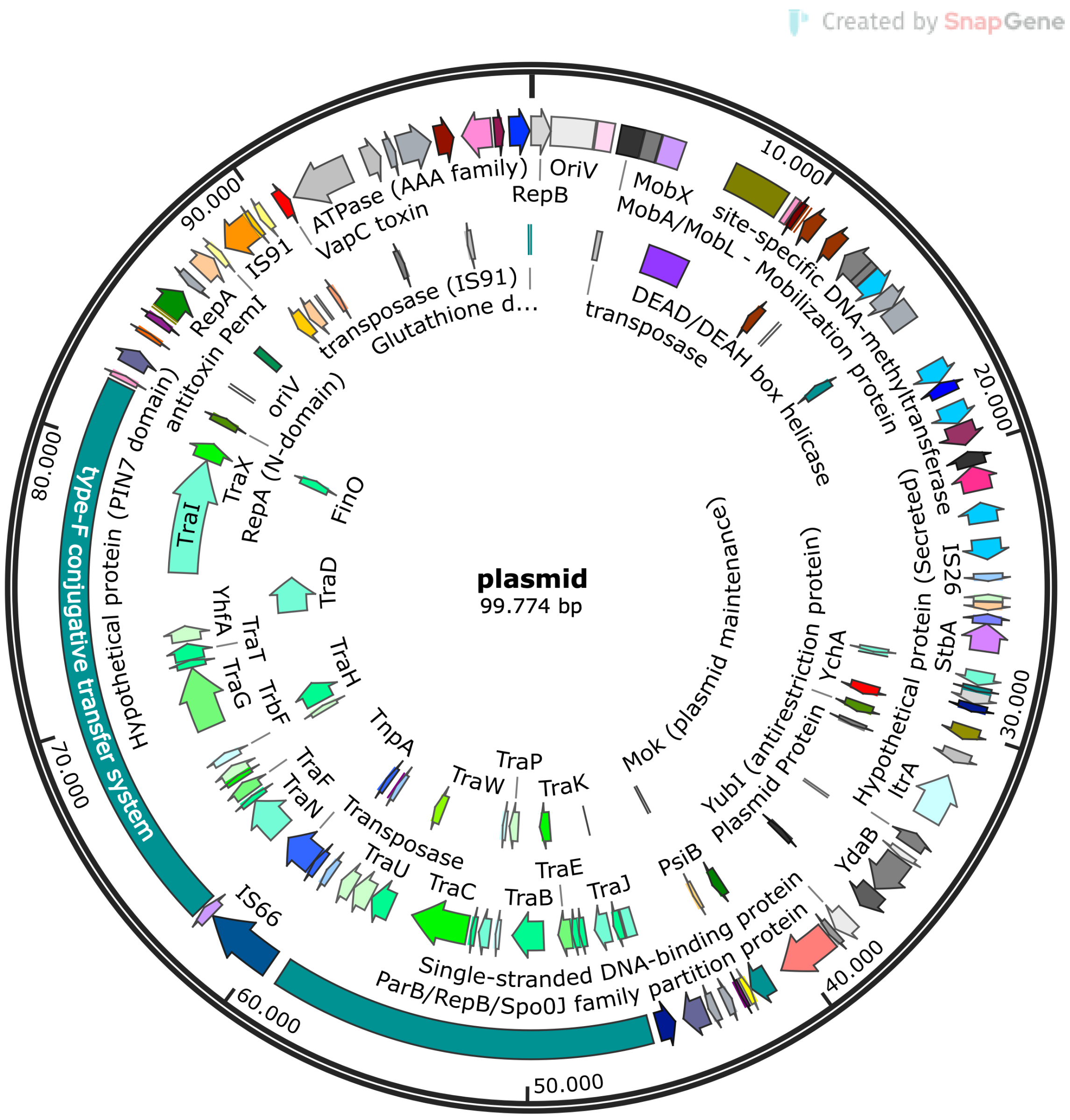

Supplementary Figure 7. Annotated map of the study plasmid using PATRIC, PROKKA and BAKTA for annotations and SnapGeneViewer to reconstruct the map.

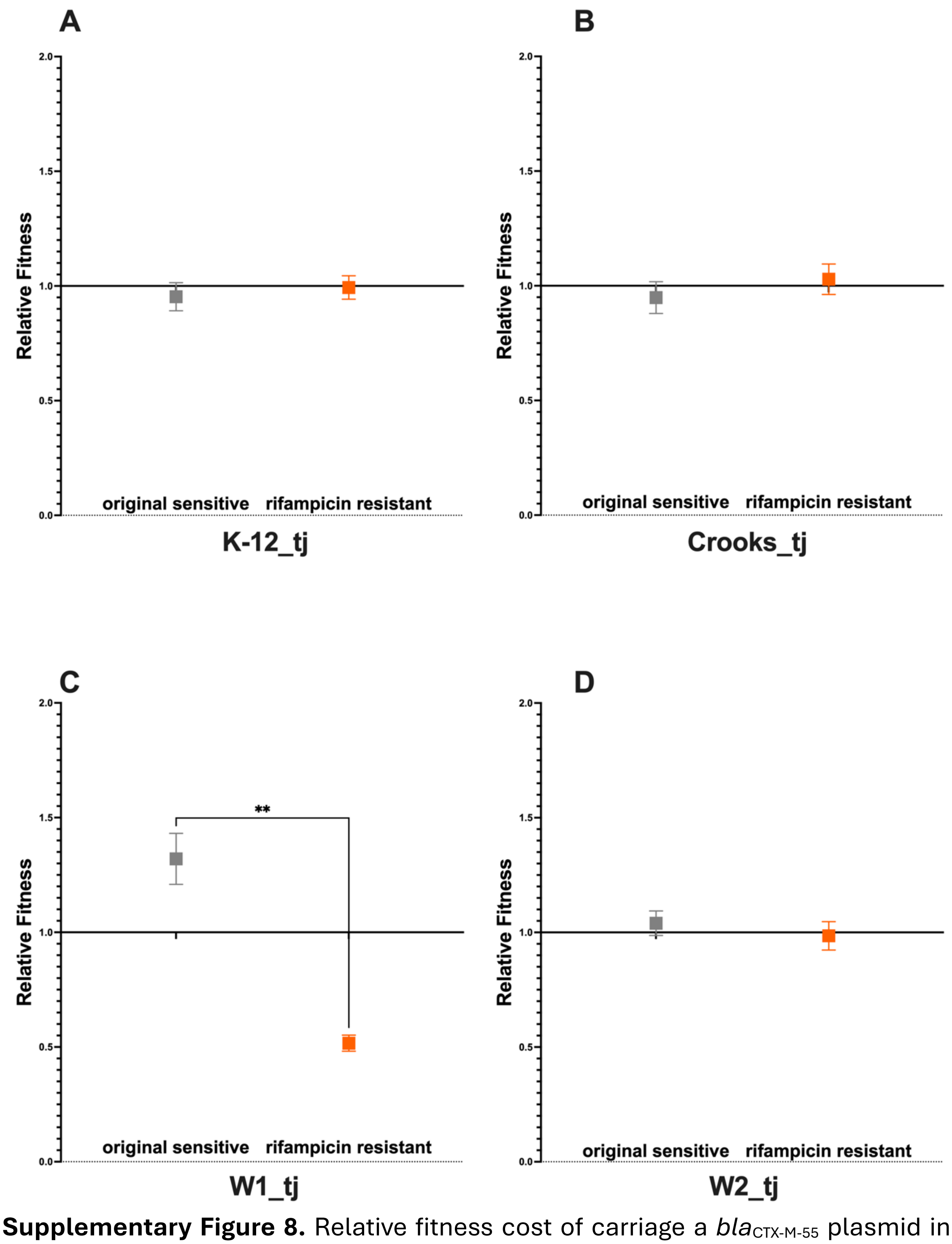

Supplementary Figure 8. Relative fitness cost of carriage a *bla*$_{CTX-M-55}$ plasmid in the strains sensitive to rifampicin and resistant to rifampicin in DM medium. Asterisks represented Mann-Whitney test results with p values less than or equal to 0.05. **A** corresponds to the relative fitness of strains K-12_tj and K-12^{rr}_tj in DM, **B** corresponds to the relative fitness of strains Crooks_tj and Crooks^{rr}_tj in DM, **C** corresponds to the relative fitness of strains W1_tj and W1^{rr}_tj in DM, and **D** corresponds to the relative fitness of strains W2_tj and W2^{rr}_tj in DM.

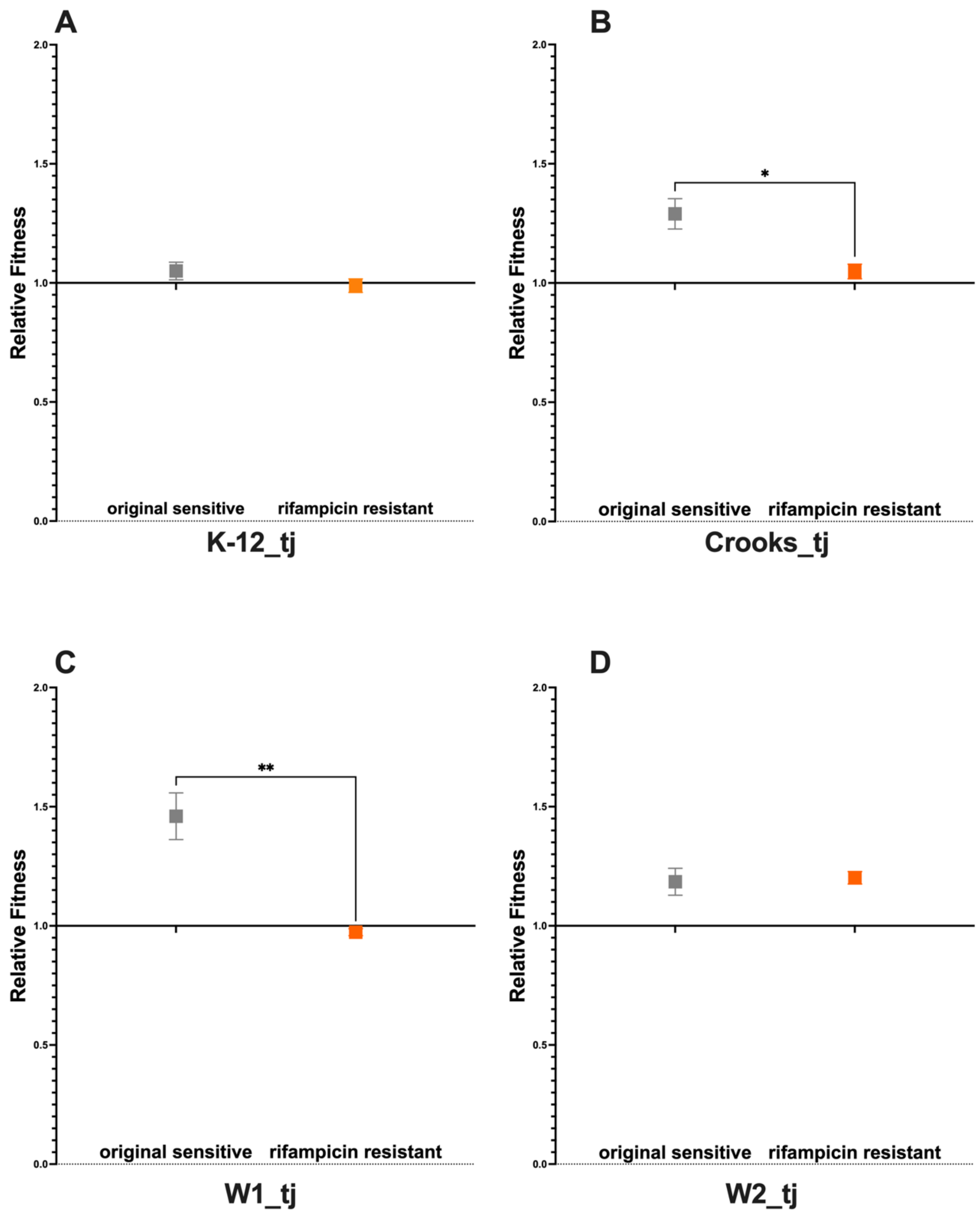

Supplementary Figure 9. Relative fitness cost of carriage a *bla*$_{CTX-M-55}$ plasmid in the strains sensitive to rifampicin and resistant to rifampicin in LB medium. Asterisks represented Mann-Whitney test results with p values less than or equal to 0.05. **A** corresponds to the relative fitness of strains K-12_tj and K-12^{rr}_tj in LB, **B** corresponds to the relative fitness of strains Crooks_tj and Crooks^{rr}_tj in LB, **C** corresponds to the relative fitness of strains W1_tj and W1^{rr}_tj in LB, and **D** corresponds to the relative fitness of strains W2_tj and W2^{rr}_tj in LB.

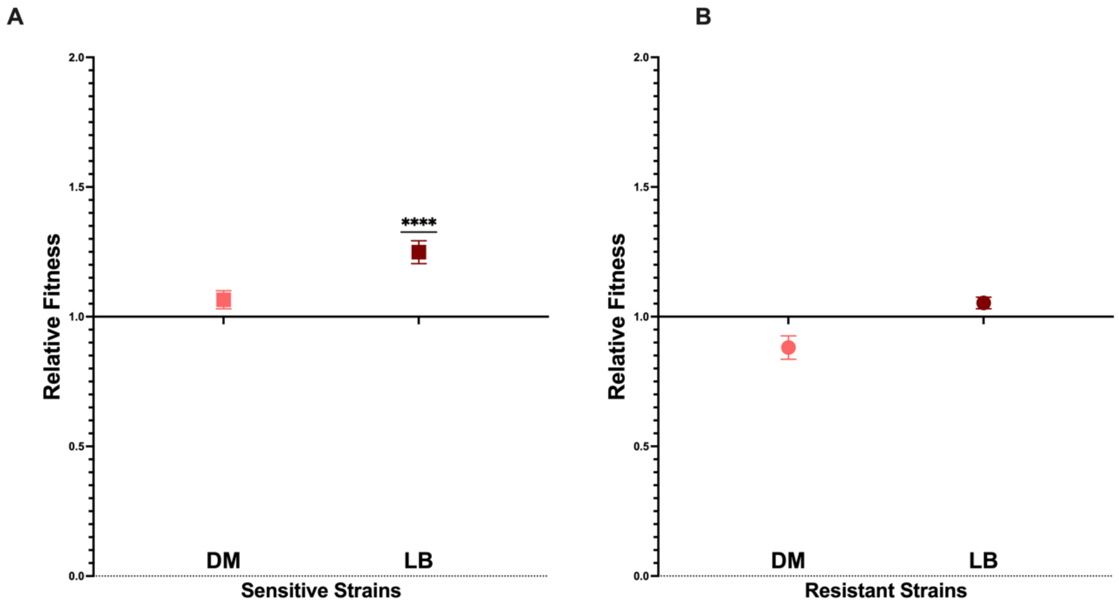

Supplementary Figure 10. Relative fitness grand mean of carriage a *bla*$_{CTX\text{-}M\text{-}55}$ plasmid for all our *E. coli* strains assayed in DM medium (light pink) and LB medium (brown). For the parenteral sensitive strains (**A**) and their resistant descendants (**B**). Asterisks represented one sample Wilcoxon test results with $p \leq 0.05$.
